# Immunological Effects of Oenothein B, an Ellagitannin Dimer, on Dendritic Cells

**DOI:** 10.3390/ijms14010046

**Published:** 2012-12-20

**Authors:** Morio Yoshimura, Hiroshi Akiyama, Kazunari Kondo, Kozue Sakata, Hideki Matsuoka, Yoshiaki Amakura, Reiko Teshima, Takashi Yoshida

**Affiliations:** 1 College of Pharmaceutical Sciences, Matsuyama University, 4-2 Bunkyo-cho, Matsuyama, Ehime 790-8578, Japan; E-Mails: myoshimu@cc.matsuyama-u.ac.jp (M.Y.); tyoshida@gem.e-catv.ne.jp (T.Y.); 2 National Institute of Health Sciences, 1-18-1 Kamiyoga, Setagayaku, Tokyo 158-8501, Japan; E-Mails: akiyama@nihs.go.jp (H.A.); kondo@nihs.go.jp (K.K.); sakata@nihs.go.jp (K.S.); hideki_matsu@livedoor.com (H.M.); rteshima@nihs.go.jp (R.T.)

**Keywords:** dendritic cell, oenothein B, epigallocatechin gallate, cytokine, caspase

## Abstract

Oenothein B is a unique macrocyclic ellagitannin dimer that has been found in various medicinal plants belonging to Onagraceae, Lythraceae, and Myrtaceae, with diverse biological activities. The immunological effects of tannins in terms of cytokine-release from macrophages and monocytes have been discussed, while the effects on other immunocompetent cells have been the subject of minimal investigation. We evaluated the immunomodulatory effects induced by tannin treatment in human dendritic cells (DCs), which play a critical role in the initial immune response, by measuring the changes in cytokine production, cell differentiation, and cell viability. Oenothein B showed significant down-regulation of the expression of cell surface molecules, CD1a and CD83, suggesting the inhibition of DC differentiation and/or maturation. The suppressive effect on DCs was associated with the induction of apoptosis without the activation of caspase-3/7, 8, and 9, and this was supported by the morphological features indicating significant nuclear condensation. Oenothein B also markedly suppressed the production of inflammatory cytokines, such as IL-1β and IL-6, in a dose-dependent manner. These data may, in part, be able to explain the traditional use of tannin-containing medicinal plants for the treatment of a variety of inflammatory diseases, including inflammatory bowel disease, celiac disease, and rheumatoid arthritis.

## 1. Introduction

Polyphenols, widely distributed in medicinal plants, foods and beverages, have currently been attracting great interest because of their diverse biological properties, including potent antioxidative effects, which are beneficial to human health. Among such bioactive polyphenols are ellagitannins, classified as large molecular weight tannins and found in many traditional medicines in Japan and China, and which have been demonstrated to exhibit inhibitory effects on various enzymes, anti-tumor and antimicrobial activities, as well as antioxidative effects [1]. The biological activities of tannins also include a number of effects associated with immunomodulation, such as host-mediated antitumor effects by oenothein B and other dimeric ellagitannins [2], anti-leishmanial activity by geraniin and other dehydroellagitannins [3] and antitumor-promoting effects by geraniin and a green tea tannin, EGCG [4].

**Figure 1:**
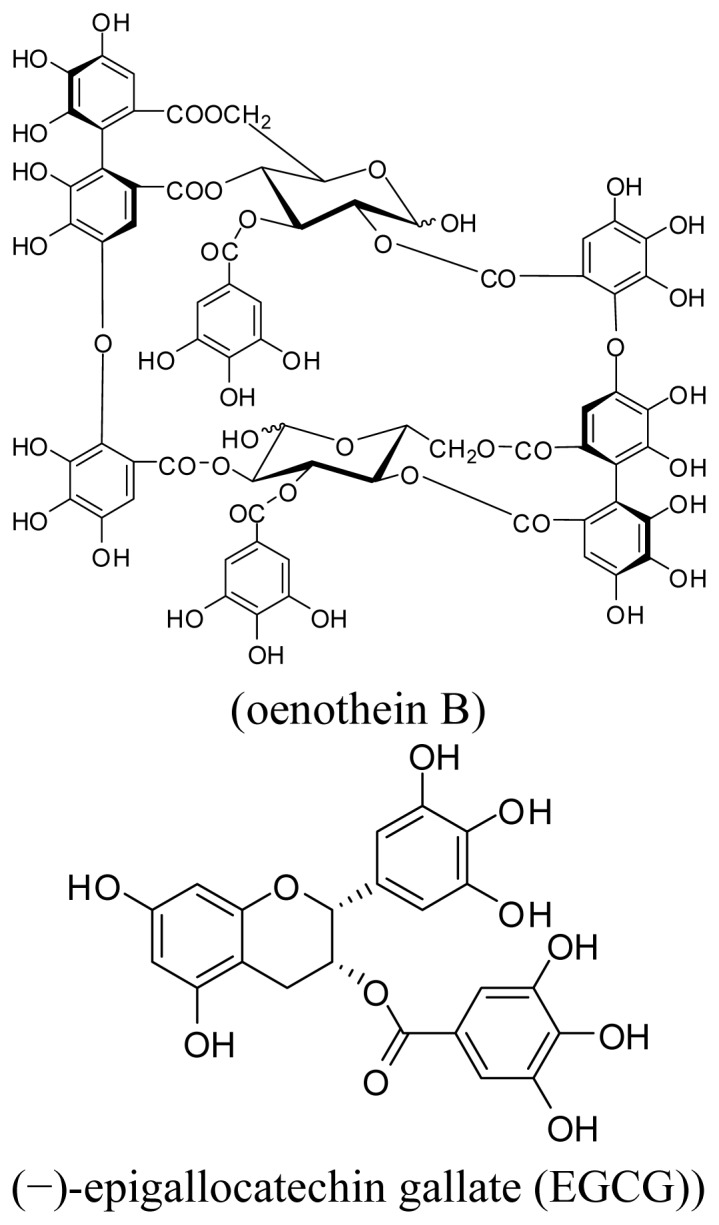
Structures of oenothein B and EGCG.

Among tannins, oenothein B is a unique macrocyclic ellagitannin dimer (Figure 1) that was first isolated from *Oenothera erythrosepala* (Onagraceae) [5] and later found widely distributed in various medicinal plants belonging to Onagraceae, Lythraceae [6] and Myrtaceae [7]. Notably its host-mediated antitumor activity against a model cancer cell line, sarcoma 180 was the most potent among over a hundred tannins and related polyphenols examined. This effect was attributed to the activation of macrophage-releasing IL-1β [2]. Meanwhile, the suppressing effect of IL-1β and IL-6 secretion by oenothein B associated with anti-inflammatory effects was also reported in recent study [8]. Other immunomodulatory effects of tannins have also been discussed, mostly in terms of cytokine-release from macrophages and monocytes [9], while the effects of tannins on other immunocompetent cells have been the subject of minimal investigation to date.

Among the immune cells, DCs are bone marrow-derived leucocytes and play a critical role in the initial immune response. Immature DCs have strong phagocytic ability, and after antigen uptake and maturation, matured DCs migrate to lymph nodes and present antigens to naive T cells. DCs, macrophages and B cells are involved in antigen presentation with MHC class II molecules and therefore these cells are called professional antigen-presenting cells. Another important function of cytokine production inherent to DCs is its constitutive role in initiating inflammation related to some autoimmune diseases, including inflammatory bowel disease, celiac disease [10] and rheumatoid arthritis [11].

In the present study, we assessed the effects of oenothein B on human DCs by measuring changes in cytokine production, cell differentiation, and cell viability, thereby enhancing our understanding of tannins in medicinal plants.

## 2. Results

### 2.1. Analysis of Cell Apoptosis Using Flow-Cytometry

**Figure 2:**
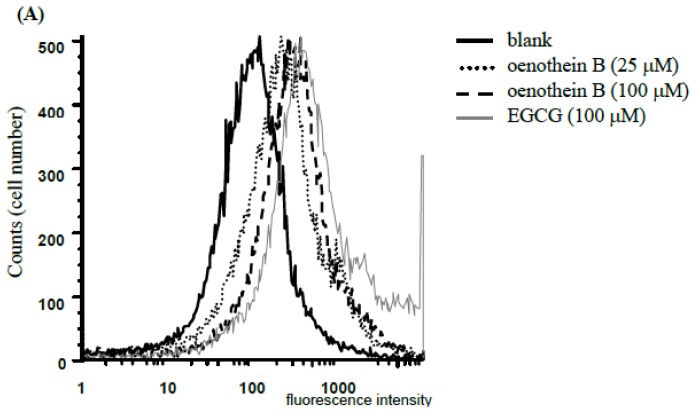

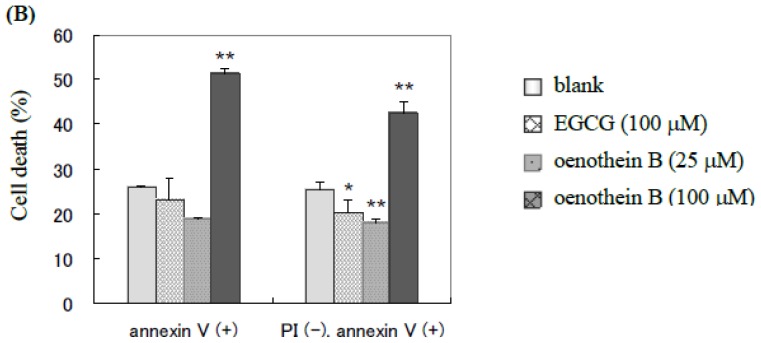
Identification of the type of cell death. iDCs were seeded at a density of 3.0 × 10^5^ cells per well in 12-well plates, and cultured with EGCG (100 μM), oenothein B (25, 100 μM) or culture medium (blank) for 22 h supplemented with TNF-α (75 ng/mL) and LPS (100 ng/mL). (**A**) Cells were harvested and stained with propidium iodide (PI) before flow-cytometric analyses. PI-stained cells were increased with tannin treatment and the cells were presumed to be necrotic cells; (**B**) Cells were stained with PI and annexin V, and analyzed by flow-cytometry. Apoptotic ratio of DCs was estimated by double-staining with PI and annexin V. Each data point represents the mean ± SD, *n* = 3, for *p < 0.05, **p < 0.01 *vs.* blank group (□) using the student’s *t*-test.

EGCG is known to induce cell death in an apoptotic (≤50 μM) or necrotic (>50 μM) manner [12]. To evaluate the effects of oenothein B on DCs, we analyzed the cells stained with PI and Annexin V using flow-cytometry. The cells stained with PI were taken as dead cells (Figure 2A), and PI (−) and annexin V (+) cells were considered as apoptotic cells (Figure 2B).

**Figure 3:**
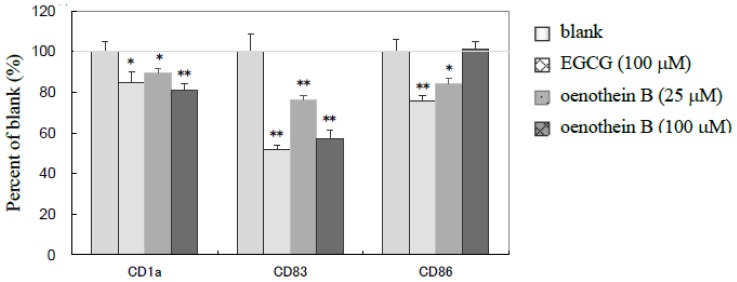
Changes in the expression of cell surface molecules. iDCs were cultured with EGCG (100 μM), oenothein B (25, 100 μM) or culture medium (blank) for 22 h at a density of 3.0 × 10^5^ cells per well in 12-well plates supplemented with TNF-α (75 ng/mL) and LPS (100 ng/mL). Cells were treated with fluorescence-labeled monoclonal antibodies against CD1a, CD83 and CD86 for flow-cytometric analysis, and the expression of cell surface molecules were determined. Each data point represents the mean ± SD, *n* = 3, for * *p* < 0.05, ** *p* < 0.01 *vs.* blank group (□) using the student’s *t*-test.

As shown in Figures 2 and 3, treatment with 100 μM oenothein B specifically induced the cell death and apoptosis of cultured DCs, whereas EGCG-treatment (100 μM) did not induce apoptosis to a greater extent than that of control. On the other hand, low-dose treatment (25 μM) of oenothein B reduced the number of apoptotic cells, although the ratio of total PI (+) cells was slightly higher than that of the non-treatment group.

### 2.2. Flow-Cytometric Analyses of Cell Surface Molecules

The cultured cells were stained with fluorescence-labeled monoclonal antibodies against CD1a, CD83, CD86, and analyzed using flow-cytometry. The cell surface molecules of CD1a and CD83 were down-regulated by oenothein B or EGCG, while CD86 was not significantly changed (Figure 3) upon treatment with oenothein B (100 μM). As CD83 expression is known to be a marker of matured DCs [13] and to play a critical role in antigen presentation, that the tannins studied implied that tannins are inhibitors of iDC differentiation and/or maturation.

### 2.3. Quantification of Cytokines in Cell Culture Medium Supernatant

**Figure 4:**
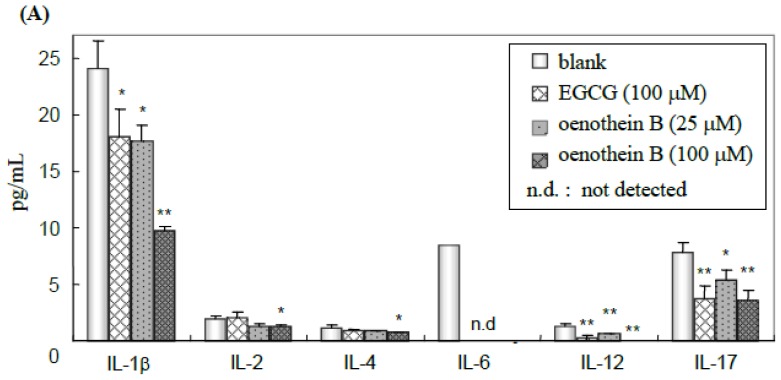

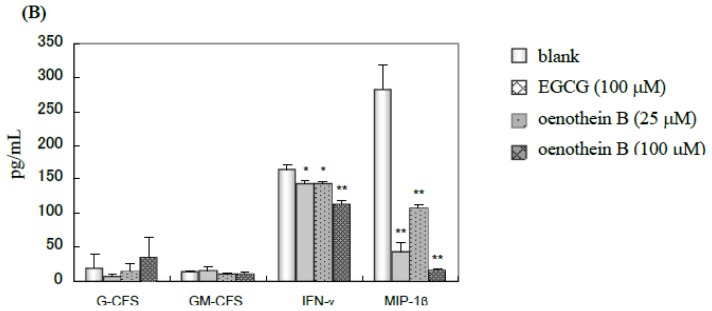
Cytokine production upon treatment with tannins. Cells were cultured in the presence of EGCG (100 μM), oenothein B (25, 100 μM) or culture medium (blank) at a density of 1.4 × 10^5^ cells per well in 12-well plates for 22 h supplemented with TNF-α (75 ng/mL) and LPS (100 ng/mL). Each cultured medium was collected and applied to a Bio-Plex multiple suspension array kit (Bio-Rad) for cytokine quantification [IL-1β, 2, 4, 5, 6, 7, 8, 10, 12, 13, 17, granulocyte colony stimulating factor (G-CSF), granulocyte macrophage colony stimulating factor (GM-CSF), interferon (IFN)-γ, monocyte chemotactic protein (MCP)-1, macrophage inflammatory protein (MIP)-1β and tumor necrosis factor (TNF)-α]. (**A**) Quantification of cytokines (IL-1β, 2, 4, 6, 12, and 17) in tannin-treated cell culture medium; (**B**) Quantification of cytokines (G-CSF, GM-CSF, IFN-γ, and MIP-1β) in tannin-treated cell culture medium. Each data point is expressed as percentage relative with blank group and represented the mean ± SD, *n* = 3, for *p < 0.05, **p < 0.01 *vs.* blank group (□) using the student’s *t*-test.

The cell culture supernatants were analyzed using a Bio-Plex multiple suspension array kit to quantify 17 cytokines (IL-1β, 2, 4, 5, 6, 7, 8, 10, 12, 13, 17, G-CSF, GM-CSF, IFN-γ, MCP-1, MIP-1β and TNF-α). The parameters significantly changed compared to blank are shown in Figure 4. Cytokine productions of IL-1β, IL-6, IL-12, IL-17, IFN-γ and MIP-1β were down-regulated in a dose-dependent manner by oenothein B-treatment, and the efficacy of oenothein B was more potent than that of EGCG. Furthermore, the inflammatory cytokines IL-1β and IL-6 were significantly down-regulated. These results suggest that these tannins may have anti-inflammatory effects through the inhibitory effects of DC inflammatory cytokine production.

### 2.4. Measurement of Caspase Activities

**Figure 5:**
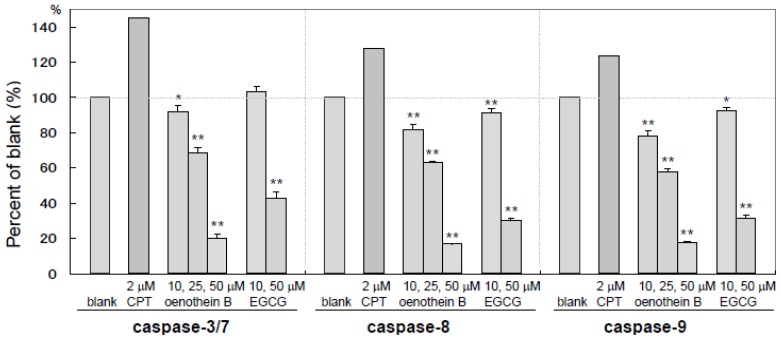
Caspase activities in tannin-treated DCs. Cells were cultured at a density of 2.3 × 10^5^ cells per well in 12-well plates for 22 h in the presence of camptothecin (CPT), EGCG (10, 50 μM), oenothein B (10, 25, 50 μM) or culture medium (blank) supplemented with TNF-α (75 ng/mL) and LPS (100 ng/mL). Each cell lysate was prepared using Reporter Lysis Buffer (Promega), and the caspase-3/7, -8 and -9 activities were measured by the respective caspase assay kits (Promega). Each data point is expressed as percentage relative with blank group and represented the mean ± S.D., *n* = 3, for *p < 0.05, **p < 0.01 *vs.* blank group (□) using the student’s *t*-test.

Caspases play an important role in cell apoptosis and are composed of several sub-types with individual functions. Caspase-3 and 7 are known to act as effectors of apoptosis and caspase-8 and 9 are initiators of apoptosis. As shown in Figure 5, caspase-3/7, 8 and 9 activities of cultured DCs supplemented with oenothein B or EGCG were dose-dependently and significantly down-regulated. While CPT, which is a well-known apoptosis-inducing compound, activated these enzymes as anticipated. These results show that these tannins preferentially inhibit the activation of caspase-3/7, 8 and 9. The findings suggest that induction of cell death with tannin treatment occurred in dose-dependent and caspase-independent manners.

### 2.5. Morphological Analysis of Tannin-Treated DCs

**Figure 6:**
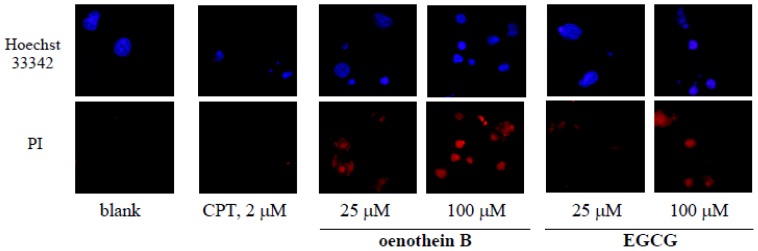
Fluorescence microscopy observations of tannin-treated DCs. Cultured cells (3.3 × 10^5^ cells per well in 12-well plates for 24 h) treated with EGCG (25, 100 μM), oenothein B (25, 100 μM) or culture medium (blank) were fixed to CELL-TAK (BD Biosciences) coated 8-well chamber slides. After staining with Hoechst 33342 and PI, cells were mounted with ProLong Gold Antifade Reagent (Invitrogen, Carlsbad, CA, USA) and evaluated under a fluorescence microscope (×400).

Cultured DCs were stained with PI and Hoechst 33342, and nuclear fragmentation or other features were evaluated under a fluorescence microscope (×400). DCs treated with oenothein B or EGCG showed significant reduction of nuclear size, in contrast with cell nuclear fragmentation in the CPT-treated group as positive control (Figure 6). These morphological features resemble AIF (apoptosis-inducing factor)/PARP (poly (ADP-ribose) polymerase)-dependent cell death [14]. These results suggest that oenothein B and EGCG affect nuclear size, not cell nuclear fragmentation as observed with CPT.

## 3. Discussion

We investigated the influence of tannins on DC associated immune responses, and found that oenothein B and EGCG have immunoregulatory effects on DCs through suppression of cell surface molecules, down-regulation of cytokine production and induction of their apoptosis.

The inhibitory effect of these tannins on the expression of cell surface molecules CD1a, CD83 and CD86 indicated they would induce the dysfunction of DC-mediated immune responses by the inhibition of cell maturation and subsequent antigen presentation. Furthermore, for cytokine production, IL-1β, 6, 12, 17, IFN-γ and MIP-1β were dose-dependently down-regulated, whereas other cytokines, IL-2, 4, G-CSF, GM-CSF, and IL-5, 7, 8, 10, 13, MCP-1, TNF-α (data not shown), displayed no significant change. The regulating effect of oenothein B on the cytokines was more potent than EGCG at the identical concentration. IL-1β and IL-6 are inflammatory cytokines [15]. Although oenothein B was reported to accelerate the production of inflammatory cytokines from monocytes [9], significant suppression of IL-6 production below the level of detection and down-regulation of IL-1β might thus induce the anti-inflammatory effect of tannins through DCs.

For cell viability evaluated by flow-cytometric assays, the higher increase in amount of PI stained cells in the tannin-treated groups indicated that tannins induced cell death. Though the increase in PI (−) and annexin V (+) cells upon treatment with a high concentration of oenothein B (100 μM) indicated potent induction of cell apoptosis, oenothein B (25 μM) and EGCG (100 μM) showed less severe apoptotic features. To investigate the mechanism of cell death, we examined the effects on caspases (caspase-3/7, 8 and 9), which play crucial roles in cell apoptosis. Caspase-3/7 and 8/9 are known to act as effectors and initiators of apoptosis, respectively. As shown in Figure 5, none of these caspases were activated but, instead, down-regulated dose-dependently by the tannins, suggesting that the apoptosis mechanisms were not caspase-dependent. Though EGCG was reported to induce caspase-dependent apoptosis in normal rat kidney interstitial fibroblast cells at a low concentration (≤50 μM) [12], unexpectedly, it showed no caspase activation of DCs (Figure 5) in our study. The mechanisms of induced-apoptosis with or without caspase thus appear to differ according to cell type. The caspase-independent apoptosis in tannin-treated DCs might be related to morphological changes showing nuclear condensation without DNA fragmentation, which is a characteristic feature of caspase-dependent apoptosis (Figure 6). In addition, necrosis-like cell expansion and DNA smearing were not observed in the tannin-treated cells. The findings suggest that these features are similar to AIF/PARP-dependent cell apoptosis. Further multidirectional approaches to elucidating their mechanisms should be investigated.

## 4. Experimental Section

### 4.1. Cell Line, Chemicals and Biochemicals

EGCG was purchased from Nakahara Kagaku (Gifu, Japan). Purified LPS (*Escherichia coli*; serotype O26: B6) was obtained from Sigma (St. Louis, MO, USA) and dissolved in endotoxin-free water. iDCs and culture medium ACS-100 were purchased from NEMOD GmbH & Co. (Berlin, Germany), and all cultures were performed in ACS-100. Recombinant human TNF-α and DNase I were obtained from PeproTech Inc. (Rocky Hill, NJ, USA). PI, annexin V, fluorescence (APC, FITC, PE)-labeled monoclonal antibodies (to CD1a, 83, 86) and CELL-TAK were purchased from BD Biosciences (Two Oak Park, MA, USA). CPT, Triton X-100 and paraformaldehyde phosphate buffer were purchased from Wako Pure Chemical Industries, Ltd. (Osaka, Japan). Hoechst 33342 and PI for nuclear staining were purchased from Molecular Probes (Eugene, OR, USA). Prolong Gold Antifade Reagent was obtained from Invitrogen (Carlsbad, CA, USA). Oenothein B was isolated from the leaves of *Eucalyptus globulus* as reported previously [16].

### 4.2. Cell Culture

Frozen iDCs were rapidly thawed in a water bath (37 °C), and culture medium composed of ACS-100 with DNase I (20 U/mL) was added to a final volume of 50 mL. After centrifugation (7 min, 200 × *g*) and removal of the culture medium, cells were re-suspended in ACS-100 supplemented with TNF-α (75 ng/mL) and LPS (100 ng/mL). Cells were further cultured at a density of 3.0 × 10^5^ cells per well in 12-well plates, supplemented with EGCG (10, 50, 100 μM), oenothein B (10, 25, 50, 100 μM) or culture medium (blank), for 22 h at 37 °C in a 5% CO_2_ air environment. For measurements of caspase activity and morphological changes, cells were cultured for 24 h using CPT (2 μM) as a positive control. After culturing, cells were collected and used for flow cytometric analyses, measurement of caspase activities and fluorescence microscopy (Olympus IX71, Olympus, Tokyo, Japan). The supernatants were used for cytokine assays.

### 4.3. Flow Cytometric Analyses

Cultured iDCs were collected and stained with PI and annexin V (for analysis of cell apoptosis) or fluorescence (APC, FITC, PE)-labeled monoclonal antibodies (to CD1a, 83, 86, for analysis of cell surface molecules) at concentrations indicated by the manufacturers. Data were collected using a FACSCalibur flow cytometer (Becton Dickinson, and Company, Franklin Lakes, NJ, USA) and analyzed with BD CellQuestTM software.

### 4.4. Quantification of Cytokines

Cell culture medium supernatants were analyzed using a Bio-Plex multiple suspension array kit (Bio-Rad Laboratories, Hercules, CA, USA) and 17 cytokines were quantified [IL-1β, 2, 4, 5, 6, 7, 8, 10, 12, 13, 17, G-CSF, GM-CSF, IFN-γ, MCP-1, MIP-1β and TNF-α]. Data were collected and analyzed using a Bio-Plex suspension array system.

### 4.5. Measurement of Caspase Activities

Caspase activities were assayed using a Apo-ONE Homogeneous caspase-3/7 kit, Caspase-Glo 8 assay kit and Caspase-Glo 9 assay kit (Promega, Madison, WI, USA). Reporter Lysis Buffer (Promega, Madison, WI, USA) was added to the cultured cells and freeze-thawed for cell lysis, and the homogenates were used for measurement of caspase activities. Each cell lysate was transferred to a 96-well microplate and Apo-ONE Homogeneous Caspase-3/7 Reagent (Promega, Madison, WI, USA) or Caspase-Glo 8 (or 9) Reagent (Promega) was added. After incubation at room temperature for 1 h, fluorescence for Caspase-3/7 and light emissions for Caspase-8 or 9 were measured according to the methods indicated by the manufacturers.

### 4.6. Observation of Morphological Changes

Cultured cells were collected and washed twice with PBS (−), and transferred to a CELL-TAK coated 8-well culture slide and left for 1 h to allow for cell adhesion. After removal of PBS (−), the cells were fixed with 4% paraformaldehyde for 30 min, and permeabilized with 0.2% Triton X-100 for 10 min.

After blocking with 2% BSA, the cells were stained with PI and Hoechst 33342. Finally, air-dried slides were mounted with ProLong Gold Antifade Reagent. Each slide was evaluated under a fluorescence microscope (×400).

## 5. Conclusions

The present study demonstrated that tannins exert peripheral anti-inflammatory effects by down-regulation of cytokines and induction of dysfunction and apoptosis in DCs. These effects might be important especially in digestive organs, since tannins are generally considered to be stable in acidic conditions and thus could travel without modification through the pharyngeal tube and stomach until their metabolism in the small intestine [17]. These data may, in part, be able to explain the traditional use of tannin-containing medicinal plants for the treatment of a variety of inflammatory diseases.
